# Case Report: Bilateral Choroidal Neovascular Membranes in a Patient With a Delayed Diagnosis of Acute Syphilitic Posterior Placoid Chorioretinopathy

**DOI:** 10.3389/fopht.2022.890872

**Published:** 2022-06-20

**Authors:** Sarah Schimansky, Tomás Burke

**Affiliations:** Uveitis and Medical Retina Service, Bristol Eye Hospital, Bristol, United Kingdom

**Keywords:** acute syphilitic posterior placoid chorioretinopathy, choroidal neovascular membrane, multimodal imaging, OCT angiography, intravitreal anti-VEGF

## Abstract

We report the case of a 78-year old man with a delayed diagnosis of syphilis and an advanced phenotype of acute syphilitic posterior placoid chorioretinopathy after receiving 5 months of high dose steroids prior to anti-treponemal treatment. Bilateral choroidal neovascular membranes were present at the time of diagnosis and were successfully treated with intravitreal aflibercept, following completion of anti-treponemal therapy.

## Introduction

Acute syphilitic posterior placoid chorioretinopathy (ASPPC), a term coined by Gass (1) in 1990, is characterised by pale or yellowish circular hyperautofluorescent outer retinal lesions in the posterior pole (2, 3). It is one of the multiple possible ocular manifestations of syphilis, and may occur in both immunocompromised and immunocompetent patients (2–4). Choroidal neovascular membranes (CNVMs) occur infrequently in syphilitic chorioretinitis with most cases reportedly arising years after successful treatment of primary, secondary or tertiary syphilis (5, 6). To our best knowledge, this is the first report of bilateral submacular choroidal neovascular membranes (CNVMs) diagnosed with OCT-Angiography in a patient with bilateral acute syphilitic posterior placoid chorioretinopathy due to active syphilis.

## Case Report

A 78-year-old man was referred to our service with progressive bilateral central vision loss over 5 months for which he was initially commenced on high-dose oral steroids. His vision had improved temporarily after three doses of intravenous methylprednisolone but subsequently deteriorated again despite continuing high-dose oral steroids for a total of 5 months. At presentation his best-corrected visual acuities (BCVAs) were Hand Movements in the right eye (OD) and 1/60 in the left eye (OS). His visual symptoms were preceded by 2 months of shortness of breath, general malaise and unintentional weight loss for which he was reviewed by the respiratory and oncology teams. Clinical examination did not reveal any other clinical findings, specifically no rashes. Computed tomography of the chest demonstrated pulmonary fibrosis and liver lesions, while positive emission tomography revealed multiple disseminated hot spots. Biopsies of the cervical and axillary nodes only showed non-specific inflammatory changes. His ophthalmic history included high myopia, bilateral pseudophakia, and a right eye retinal detachment repair 18 years beforehand. Despite this, his BCVAs were 6/6 in both eyes with no evidence of myopic choroidal neovascularisation (CNV) at his last ophthalmic assessment 9 months prior to presentation. Past medical history included atrial dysrhythmia for which he had a pacemaker fitted.

**Figure 1:**
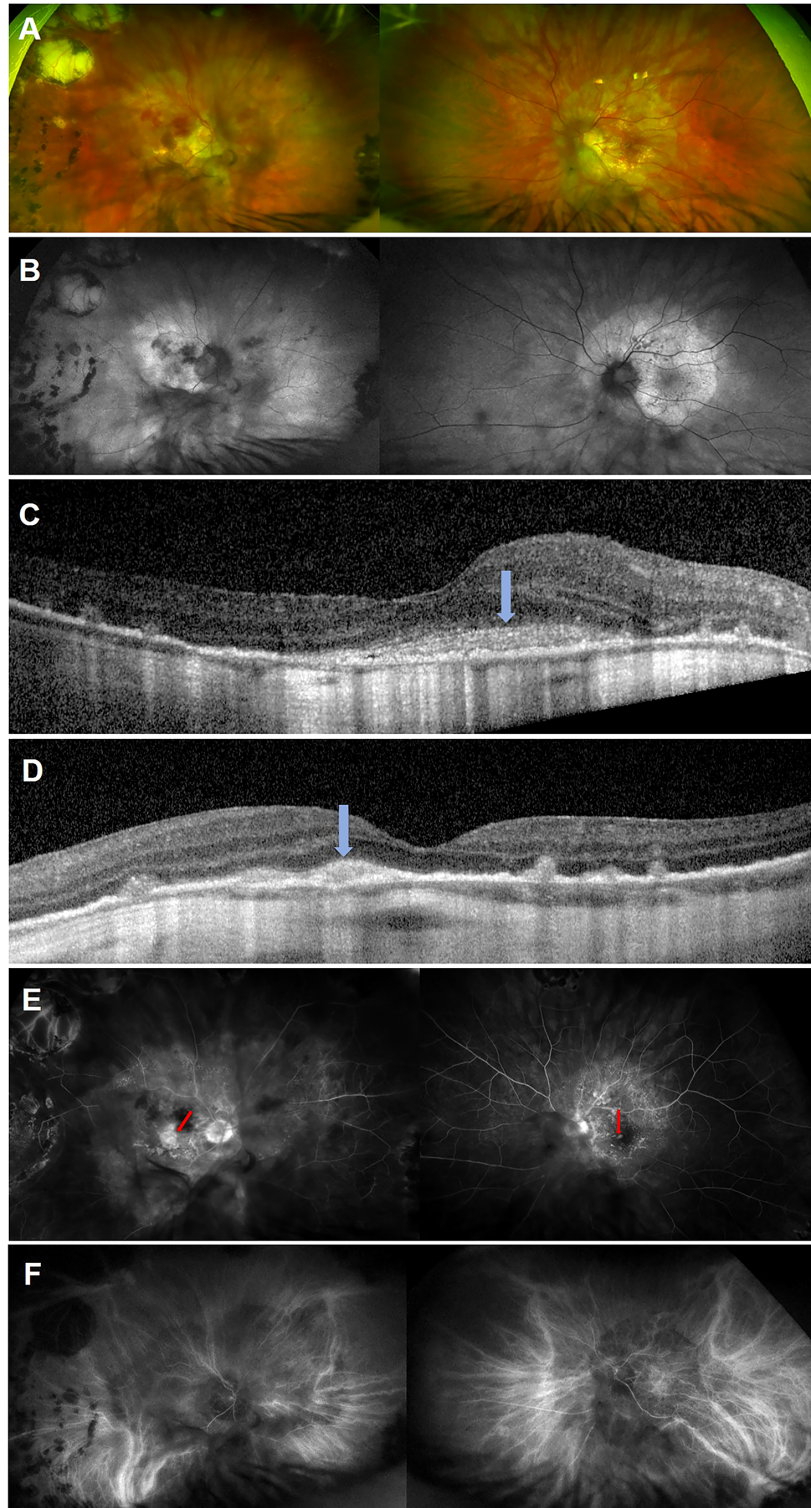
Multimodal images of both eyes taken at initial presentation. Ultra**-**widefield colour fundus photograph of both eyes **(A)** showing pale yellow placoid lesions in the posterior poles with central retinal haemorrhages (more prominent in the right eye). The right eye has temporal peripheral chorioretinal changes consistent with previous treatment of retinal detachment. Fundus autofluorescence of both eyes **(B)** demonstrating bilateral hyperautofluorescent placoid lesions with multifocal hypoautofluorescent areas of retinal pigment epithelial (RPE) atrophy (more marked in the left eye). Spectral domain-optical coherence tomography scans of the right **(C)** and left eye **(D)**, revealing disruption of the photoreceptor inner-segment ellipsoid zone, and nodular thickening of the RPE and central macular subretinal hyperreflective lesions (blue arrows) without sub-retinal fluid. Fluorescein angiography of the right eye at 1:46 minutes and left eye at 1:21 minutes **(E)** showing generalised hyperfluorescence in the region of the placoid lesions in both eyes with further window defects consistent with RPE atrophy but no evidence of widespread vasculitis. Central macular staining was visible in the right eye (red arrow), while a focal area of paracentral leak was seen in the left macula (red arrow). Indocyanine green angiography of the right eye at 5:27 minutes and left eye at 7:52 **(F)** minutes showing hypofluorescent lesions consistent with the placoid lesions in both eyes.

Slit lamp examination revealed no active anterior or vitreous inflammation and a BIO score of 0, but presence of vitreous debris bilaterally. There was extensive bilateral peripapillary and macular chorioretinopathy with few intraretinal haemorrhages (**Figure 1A**) but no overt retinal vasculitis. Fundus autofluorescence (FAF) demonstrated a distinct hyperautofluorescent placoid appearance in both eyes (**Figure 1B**) which corresponded to areas of central retinal whitening on fundus examination, suggestive of an outer retinal pathology such as syphilitic chorioretinopathy (7–9). An infective cause for this placoid fundal appearance was high on our list of differential diagnoses due to the patient’s history and non-specific multisystem symptoms. There were also multifocal areas of hypoautofluorescence consistent with retinal pigment epithelium (RPE) atrophy within the placoid lesions. Spectral domain-optical coherence tomography (SD-OCT) revealed disruption of the photoreceptor inner segment ellipsoid zones and nodular thickened irregularities of the RPE. Subretinal hyperreflective lesions were noted in the macular regions bilaterally (right > left), though without any overt intraretinal or subretinal fluid (SRF) (**Figures 1C, D**). Fundus fluorescein and indocyanine green angiography did not show any disc leak or vasculitis (**Figures 1E, F**), though the placoid lesions were clearly delineated on both modalities of angiography. Due to the presence of myopic macular degeneration and multifocal RPE atrophy, interpretation at the time of initial presentation was not felt to reveal definitive evidence active choroidal neovascularisation. During the initial review, our primary concern was an active infectious or inflammatory process and, in the absence of SRF, anti-vascular endothelial growth factor (anti-VEGF) was therefore not considered at this stage.

A complete blood count, renal and liver function, serum electrophoresis, serum angiotensin-converting enzyme, autoimmune profile, Lyme serology, Quantiferon gold test, HIV antibodies, toxoplasma IgG and IgM levels were normal or negative. Syphilis serology showed positive Syphilis IgM antibodies, positive Treponema pallidum particle agglutination (titre >1:1280) and positive rapid plasma reagin (titre 1:512). A diagnosis of bilateral ASPCC due to tertiary disseminated syphilis was made. Our patient was treated by the genitourinary medicine team with a 10-day course of intravenous penicillin, followed by 4 weeks of oral doxycycline. Oral steroids were gradually tapered.

**Figure 2:**
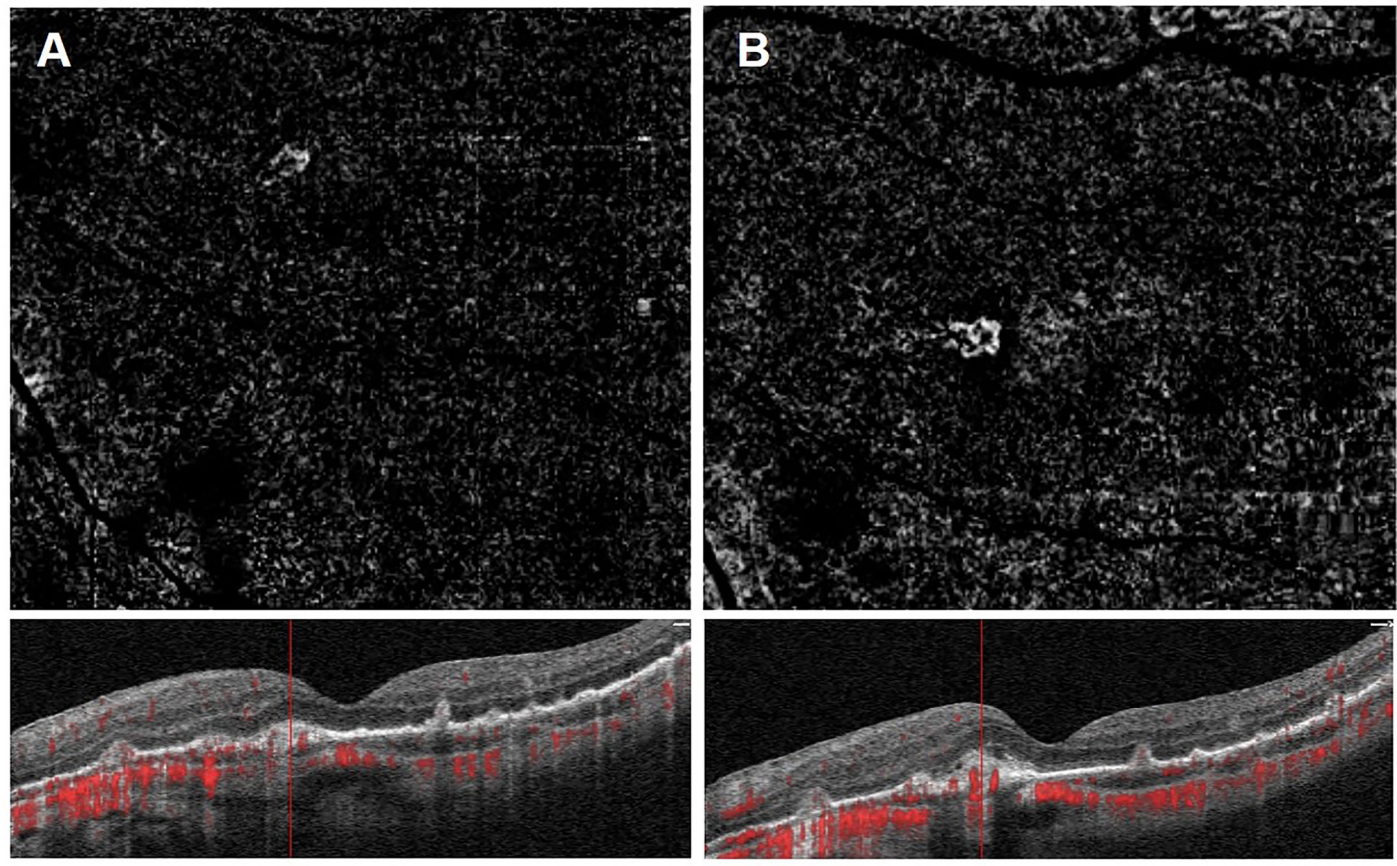
Left eye optical coherence tomography angiography of the outer retinal ‘slab’ demonstrating a choroidal neovascular membrane on the en-face image, with flow signal visible on the related B-scan at initial presentation **(A)** and revealing progression at 4-week follow-up **(B)** with subtle subretinal fluid.

Six weeks following initiation of anti-treponemal treatment, he reported symptomatic improvement in peripheral vision but persistent reduction in his central vision. At that time, his BCVAs were 6/18 OD and 6/30 OS. Interestingly, follow-up SD-OCT demonstrated new SRF bilaterally and OCT angiography (OCT-A) confirmed presence of a CNVM in the left macula (**Figure 2**). OCT-A of the right macula was not informative. Retrospective review of the baseline OCT-A (**Figure 2**), FFA and SD-OCT scans (**Figure 1**), taken together with new SRF bilaterally, was suggestive of progressing bilateral CNVMs for which bilateral intravitreal aflibercept (2mg/0.05mL) injections were commenced.

**Figure 3:**
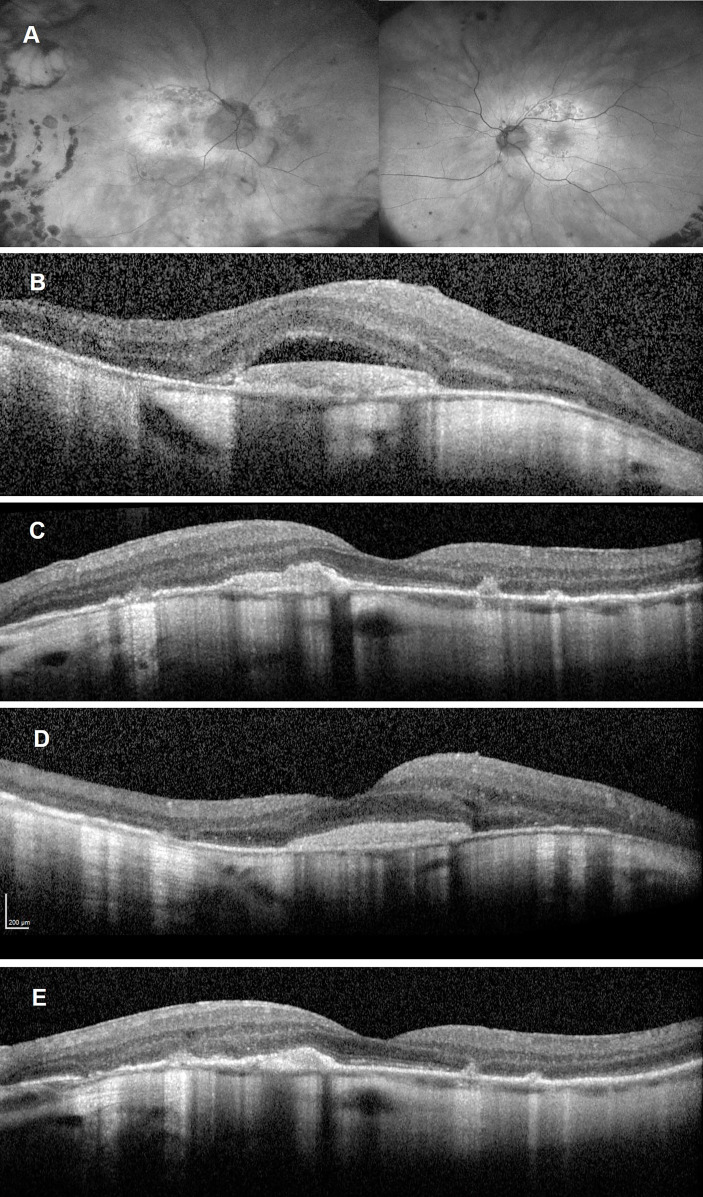
Follow-up imaging after commencement of aflibercept treatment and completion of anti-treponemal therapy. Ultra**-**widefield autofluorescence of both eyes **(A)** showing fading of the hyperreflective placoid lesion and progressing multifocal retinal pigment epithelium atrophy. Spectral Domain-Optical Coherence Tomography (SD-OCT) after one aflibercept injection showing persistent subretinal fluid overlying a disciform scar in the right eye **(B)** and resolution of subretinal fluid but persistent subretinal hyperreflective material left eye **(C)**. SD-OCT after two aflibercept injections showing a significant reduction in subretinal fluid over a largely unchanged disciform scar in the right eye **(D)** and a reduction in the subretinal hyperreflective material as well as reconstitution of the inner segment ellipsoid zone in the fovea of the left eye **(E)**.

Following two intravitreal aflibercept injections, BCVA improved to 6/12 OD and 6/6 OS. FAF demonstrated fading of the hyperautofluorescent placoid lesions in the posterior poles (**Figure 3A**). SD-OCTs showed a gradual resolution of SRF in the both eye (**Figures 3B–E**), indicating good clinical and anatomical response to aflibercept monotherapy. Syphilis serology following anti-treponemal treatment has not yet been repeated.

## Discussion

The incidence of syphilis (*Treponema pallidum*) has been increasing in the United Kingdom (10) with a similar trend observed in the United States (2). Often described as the Great Masquerador, tertiary syphilis can present with multiorgan involvement as was the case with our patient, although ocular signs and symptoms may be the initial presenting feature in up to 50% of patients (4). Ocular manifestations are varied with uveitis being present in approximately 5% of patients with tertiary syphilis (11). Multimodal imaging techniques have identified characteristic features of syphilitic chorioretinitis. Typical OCT findings in ASPPC include disruption of the external limiting membrane, nodular elevation of the OS/RPE junction and hyperreflectivity of the RPE (3, 7). While not present in our patient, the presence of inner retinal precipitates on fundus examination and SD-OCT imaging is also highly suggestive of syphilitic retinitis (12, 13).

Syphilitic chorioretinitis should be managed according to a neurosyphilis protocol with intravenous or intramuscular penicillin (2, 14). Multiple case series have demonstrated excellent visual recovery and resolution of clinical and angiographic findings with early diagnosis and treatment of the condition (2, 3, 8). However, delayed diagnosis and immunosuppression such as HIV co-infection or steroid therapy adversely affect visual prognosis (4, 15). Zamani et al. (15) described a case of ASPPC which occurred following commencement of high-dose oral steroids for presumed inflammatory chorioretinitis and cystoid macular oedema. Discontinuation of steroid therapy led to resolution of the placoid lesions and a subsequent diagnosis of syphilitic chorioretinitis. In our case, it is likely that prolonged treatment with systemic steroids and delayed commencement of anti-treponemal therapy led to sustained choroidal inflammation and a more advanced ocular phenotype. The observed development of multifocal bilateral RPE atrophy is most consistent with previous choroiditis and may have served as a trigger for the development of inflammatory-type CNVMs. This further underscores the need to exclude infectious aetiologies before commencing prolonged systemic immunosuppression in patients with uveitis. It is plausible that delayed diagnosis and treatment with systemic steroids contributed to the advanced ASPPC phenotype, leading to RPE and Bruch’s membrane atrophy that served as a trigger for choroidal neovascularisation. It is, however, possible that the patient’s age, myopia, inflammation/infection, or a combination of these could have contributed to the development of CNVM. Visual recovery to 6/12 in the right and 6/6 in the left eye following anti-VEGF treatment is more suggestive of an acute inflammatory process causing CNV, and less consistent with a more chronic degenerative process, such as myopia or age-related macular degeneration.

Our case is the first report of bilateral active CNVMs occurring in a patient with newly diagnosed active ASPPC. Previous case reports only described CNVM in cases of diffuse syphilitic chorioretinitis, occurring between 2 and 30 years after treatment of active syphilis and associated chorioretinitis (5, 6). A recent case of subfoveal CNVM in a patient with a history of syphilitic chorioretinitis was successfully treated with a combination of intravitreal anti-VEGF and oral steroids (5). Similar to the report by Giuffrè et al., we were able to identify active CNVMs using OCT-A. However, we opted for intravitreal aflibercept monotherapy, while discontinuing oral steroid treatment, in view of the history of only recently treated active syphilis with ocular involvement and the advanced phenotype related to previous steroid therapy. Our patient demonstrated an excellent response to this treatment.

In general, this case serves as a reminder to exclude infectious causes of uveitis prior to commencing systemic immunosuppression, and monitor for complications such as CNVMs in patients with ASPPC.

## Data Availability Statement

The original contributions presented in the study are included in the article/supplementary material. Further inquiries can be directed to the corresponding author.

## Ethics Statement

Ethical review and approval was not required for the study on human participants in accordance with the local legislation and institutional requirements. The patients/participants provided their written informed consent to participate in this study. Written informed consent was obtained from the individual(s) for the publication of any potentially identifiable images or data included in this article.

## Author Contributions

SS and TB were involved in clinical care of the patient, prepared and revised the manuscript. The final version of this manuscript was approved by both authors.

## Conflict of Interest

TB received personal fees from Alimera, Bayer, Novartis, Roche and Allergan, outside from and unrelated to the submitted work.

The remaining author declares that the research was conducted in the absence of any commercial or financial relationships that could be construed as a potential conflict of interest.

## Publisher’s Note

All claims expressed in this article are solely those of the authors and do not necessarily represent those of their affiliated organizations, or those of the publisher, the editors and the reviewers. Any product that may be evaluated in this article, or claim that may be made by its manufacturer, is not guaranteed or endorsed by the publisher.
